# A longitudinal analysis of immune escapes from HLA-B*13-restricted T-cell responses at early stage of CRF01_AE subtype HIV-1 infection and implications for vaccine design

**DOI:** 10.1186/s12865-022-00491-7

**Published:** 2022-04-02

**Authors:** Hui Zhang, Chuan He, Fanming Jiang, Shuang Cao, Bin Zhao, Haibo Ding, Tao Dong, Xiaoxu Han, Hong Shang

**Affiliations:** 1 NHC Key Laboratory of AIDS Immunology (China Medical University), National Clinical Research Center for Laboratory Medicine, The First Affiliated Hospital of China Medical University, 155 Nanjing North Street, Heping District, Shenyang, 110001 Liaoning Province China; 2 Key Laboratory of AIDS Immunology, Chinese Academy of Medical Sciences, Shenyang, 110001 China; 3 Key Laboratory of AIDS Immunology of Liaoning Province, Shenyang, 110001 China; 4 Collaborative Innovation Center for Diagnosis and Treatment of Infectious Diseases, 79 Qingchun Street, Hangzhou, 310003 China; 5 Department of Laboratory Medicine, The First Affiliated Hospital of China Medical University, Shenyang, 110001 China; 6 Department of Laboratory Medicine, China Medical University Shengjing Hospital Nanhu Branch, Shenyang, 110001 China; 7 Nuffield Department of Medicine, Chinese Academy of Medical Sciences Oxford Institute, Oxford University, Oxford, UK; 8 Medical Research Council Human Immunology Unit, Weatherall Institute of Molecular Medicine, John Radcliffe Hospital, Oxford University, Oxford, UK

**Keywords:** HLA-B*13, CRF01_AE, HIV-1, Epitope, Escape mutation, T-cell response

## Abstract

**Background:**

Identifying immunogens which can elicit effective T cell responses against human immunodeficiency virus type 1 (HIV-1) is important for developing a T-cell based vaccine. It has been reported that human leukocyte antigen (HLA)-B*13-restricted T-cell responses contributed to HIV control in subtype B′ and C infected individuals. However, the kinetics of B*13-restricted T-cell responses, viral evolution within epitopes, and the impact on disease progression in CRF01_AE subtype HIV-1-infected men who have sex with men (MSM) are not known.

**Results:**

Interferon-γ ELISPOT assays and deep sequencing of viral RNAs were done in 14 early HLA-B*13-positive CRF01_AE subtype HIV-1-infected MSM. We found that responses to RQEILDLWV (Nef_106–114_, RV9), GQMREPRGSDI (Gag_226–236_, GI11), GQDQWTYQI (Pol_487–498_, GI9), and VQNAQGQMV (Gag_135–143_, VV9) were dominant. A higher relative magnitude of Gag-specific T-cell responses, contributed to viral control, whereas Nef-specific T-cell responses were associated with rapid disease progression. GI11 (Gag) was conserved and strong GI11 (Gag)-specific T-cell responses showed cross-reactivity with a dominant variant, M228I, found in 3/12 patients; GI11 (Gag)-specific T-cell responses were positively associated with CD4 T-cell counts (*R* = 0.716, *P* = 0.046). Interestingly, the GI9 (Pol) epitope was also conserved, but GI9 (Pol)-specific T-cell responses did not influence disease progression (*P* > 0.05), while a D490G variant identified in one patient did not affect CD4 T-cell counts. All the other epitopes studied [VV9 (Gag), RQYDQILIEI (Pol_113–122_, RI10), HQSLSPRTL (Gag_144–152_, HL9), and RQANFLGRL (Gag_429–437,_ RL9)] developed escape mutations within 1 year of infection, which may have contributed to overall disease progression. Intriguingly, we found early RV9 (Nef)-specific T-cell responses were associated with rapid disease progression, likely due to escape mutations.

**Conclusions:**

Our study strongly suggested the inclusion of GI11 (Gag) and exclusion of RV9 (Nef) for T-cell-based vaccine design for B*13-positive CRF01_AE subtype HIV-1-infected MSM and high-risk individuals.

**Supplementary Information:**

The online version contains supplementary material available at 10.1186/s12865-022-00491-7.

## Background

Human immunodeficiency virus type 1 (HIV-1)-specific T-cell responses play an important role in the control of HIV-1 replication [1–5]. Several T-cell-based vaccine strategies, such as those based on conserved [6], mosaic [7], immunodominant [8], and subdominant [9] epitopes, are focused on identifying immunogens that can elicit protective T-cell responses. Several protective epitopes have been identified, including the human leukocyte antigen (HLA)-B*27-restricted KRWIILGLNK (Gag_263–272_, KK10) epitope [10]; the B*57-restricted TSTLQEQIAW (Gag_240–249_, TW10) epitope [11, 12]; the B*51-restricted NANPDCKTI (Gag_327–345_, NI9) epitope [13]; and the B*13-restricted RQANFLGKI (Gag_429−437,_ RI9) and HQPISPRTL (Gag_144−152_, HL9) epitopes [14]. However, the many polymorphisms among the different HIV-1 subtypes [15] and immune-escape variants driven by T-cell responses [16–18] are major obstacles for immunogen screening. Consequently, understanding the kinetics of early protective immune responses to HIV-1 and the dynamics of escape mutations are extremely important for vaccine development.

The immunodominant epitopes and variation features driven by T-cell responses are reported to be HIV-1 subtype-specific due to sequence heterogeneity [15]. HLA-B*13 is associated with delayed disease progression in C subtype HIV-1-infected African populations and the features associated with B*13-restricted T-cell responses have been studied [19]. Our previous studies showed that HLA-B*13 was the relatively common HLA-B allele ﻿in HIV-1-infected Hans in Northern China [20] and B*13 restricted HL9 and RL9 epitopes contributed to slow disease progression in B′ clade HIV-1-infected ﻿HLA-A*30-B*13-C*06-positive slow progressors [14]. However, information on early CRF01_AE subtype-related T-cell responses in B*13-positive Chinese men who have sex with men (MSM) is limited. HIV-1 transmission among MSM in China is rapidly increasing [21] and CRF01_AE has become the predominant subtype [22–25. Therefore, the aim of this study was to clarify the kinetics of B*13-restricted T-cell responses, the dynamics of viral variants within epitopes, and the correlation between T-cell responses and disease progression in CRF01_AE subtype HIV-1-infected Chinese MSM.

Fourteen HLA-B*13-positive CRF01_AE subtype HIV-1-infected Chinese MSM were recruited. The results showed that B*13-restricted RQEILDLWV (Nef_106−114_, RV9), GQMREPRGSDI (Gag_226−236_, GI11), GQDQWTYQI (Pol_487−498_, GI9), and VQNAQGQMV (Gag_135−143_, VV9) were the immunodominant epitopes. A longitudinal analysis of epitope sequencing data was undertaken, while T-cell responses and their associations with disease progression were also analyzed, as were their cross-reactivity against autologous epitope sequences. A comprehensive analysis of the B*13-restricted T-cell responses in CRF01_AE subtype-infected MSM may help with the design of T-cell-based vaccines for the target population.

## Results

### Four immunodominant HLA-B*13-restricted T-cell epitopes were identified in CRF01_AE subtype HIV-1-infected MSM

**Fig. 1:**
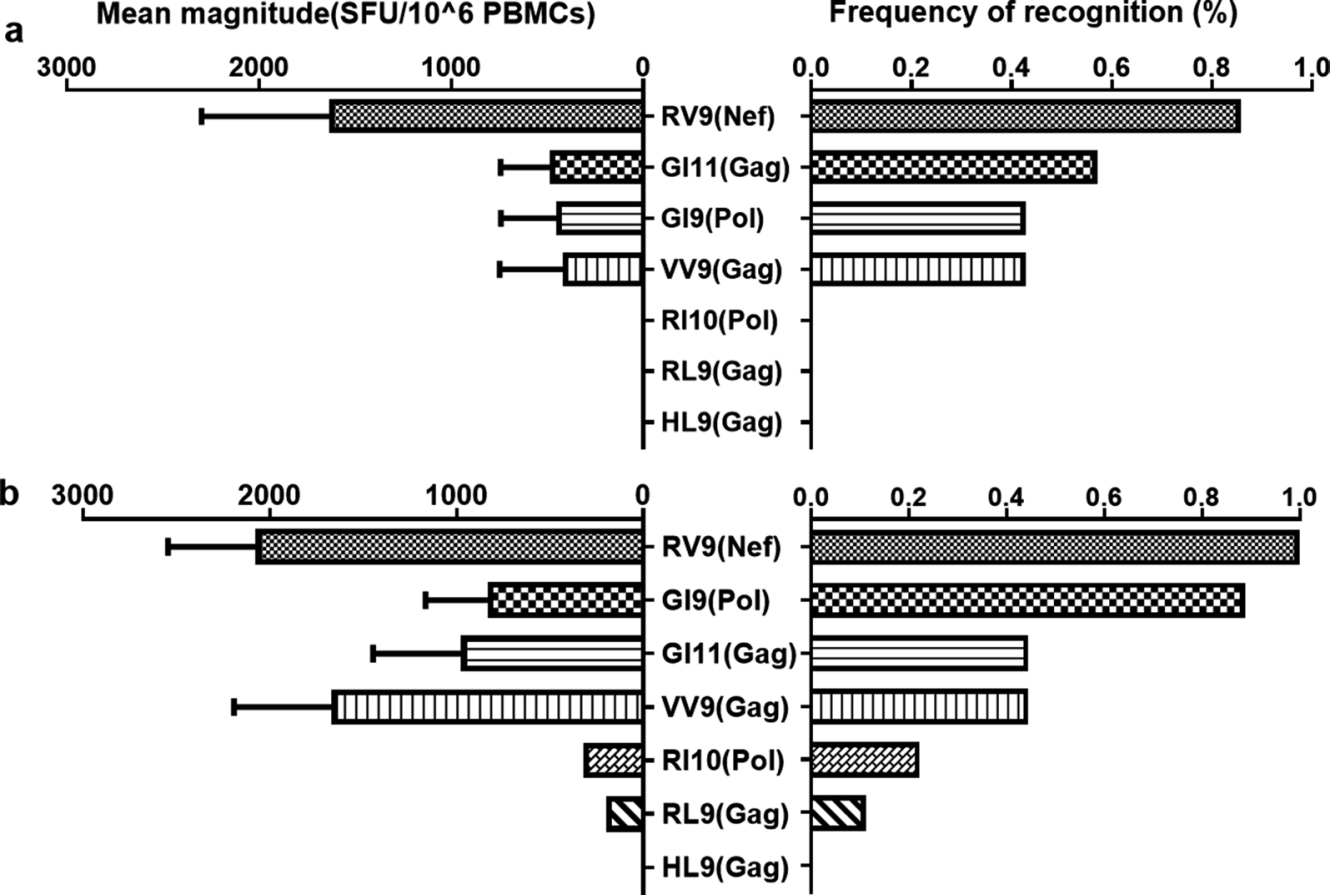
Seven HLA-B*13-restricted epitopes-specific T cell responses in CRF01_AE subtype HIV-1-infected patients. The magnitude of the response and frequency of recognition at 3 months (n = 7) (**a**) and 1 year of HIV-1 infection (n = 8) (**b**) are shown. The mean magnitudes of the responses are represented on the left, and the frequencies of recognition are displayed on the right

Fourteen HLA-B*13-positive patients were recruited in this study. The clinical details and HLA typing results of these participants are shown in Table 1. Seven HLA-B*13 epitope-specific T-cell responses in seven patients at 3 months of infection and in eight patients at 1 year of infection were detected to describe the kinetics of B*13-restricted T-cell responses. Firstly, the horizontal immunodominant epitopes, which was defined as the common reactive epitopes targeted by individuals with a specific HLA distribution at the population level [26, 27], were analyzed. Results showed that the immunodominant epitopes with a frequency of T cell responses more than 40% for this study population were RQEILDLWV (Nef_106–114_, RV9), GQMREPRGSDI (Gag_226–236_, GI11), GQDQWTYQI (Pol_487–498_, GI9), and VQNAQGQMV (Gag_135–143_, VV9) at a frequency of 85.7%, 57.1%, 42.9%, and 42.9% at 3 months of infection (Fig. 1a) and 100.0%, 44.4%, 88.9%, and 44.4% at 1 year of infection, respectively (Fig. 1b). Secondly, RV9 (Nef) elicited the strongest magnitude of responses, followed by VV9 (Gag), GI11 (Gag), and GI9 (Pol) at 1 year of infection (Fig. 1b). In addition, the breadth and magnitude of epitope-specific T-cell responses were not significantly different at 3 months of infection compared with those at 1 year of infection among six patients in whom T-cell responses were detected at both time points (*P* > 0.05, data not shown).

**Table 1:** Clinical characteristics of CRF01_AE subtype HIV-1-infected individuals

PID	HLA class I			Early			3 months^d^			1 year			Set point^a^
	HLA-A	HLA-B	HLA-C	dpi	CD4 (cells/μL)	VL (copies/mL)	dpi	CD4 (cells/μL)	VL (copies/mL)	dpi	CD4 (cells/μL)	VL (copies/mL)	
320829	A24 A24	B13 B15	C03 C03	51^b^	531	5.42	120^b^	653	5.44	352^b^	616	5.43	5.44
320018	A02 A30	B13 B13	C06 C07	34^b^	464	5.33	58^bc^	293	5.18	330^bc^	281	5.08	5.08
320853	A01 A30	B13 B40	C03 C06	43^b^	351	3.98	ND	ND	ND	226^bc^	141	4.58	4.67
321145	A11 A30	B13 B38	C06 C07	38^b^	472	5.85	138^b^	642	5.26	318^b^	525	4.79	4.62
320019	A02 A30	B13 B46	C01 C06	46^b^	163	4.46	58^bc^	163	4.61	381^bc^	411	4.77	4.50
321221	A11 A30	B13 B38	C06 C07	37^b^	549	5.32	85	548	4.70	393^b^	559	5.08	4.53
320088	A02 A11	B13 B13	C02 C03	53	ND	4.82	62^bc^	330	4.07	327^bc^	321	4.86	4.43
300471	A02 A30	B13 B35	C04 C06	134	269	4.23	139^b^	262	4.23	360^bc^	277	4.29	4.20
325020	A02 A24	B13 B40	C03 C08	ND	ND	ND	59^bc^	638	3.29	309^bc^	551	4.06	3.58
325029	A30 A30	B13 B13	C05 C06	27	429	5.40	83^b^	572	4.46	337^b^	573	3.10	3.59
320571	A02 A30	B13 B54	C01 C06	ND	ND	ND	131^c^	353	4.71	335^c^	373	4.63	4.50
320006	A02 A02	B13 B13	C03 C14	47	407	4.24	108^c^	500	3.95	395	306	3.71	3.74
320135	A02 A32	B13 B44	C03 C04	46^b^	685	3.77	63^bc^	590	4.37	427^bc^	564	3.75	3.75
440230	A02 A30	B13 B46	C01 C06	71	343	2.02	78^b^	333	4.59	371^b^	318	2.20	2.05

VL, viral load; dpi, days post-infection; ND, not detected

^a^ Average viral load (at least three time points) from 120 days to 1 year of infection

^b^ Deep-sequencing was undertaken at the indicated number of days post-HIV-1 infection

^c^ An interferon-gamma (IFN-γ) enzyme-linked immunospot (ELISPOT) assay was performed for patients at the indicated number of days post-HIV-1 infection

^d^ Available samples at the time point closest to 3 months of HIV-1 infection

### The associations of B*13-restricted Gag-, Pol-, and Nef-specific T-cell responses with disease progression

**Fig. 2:**
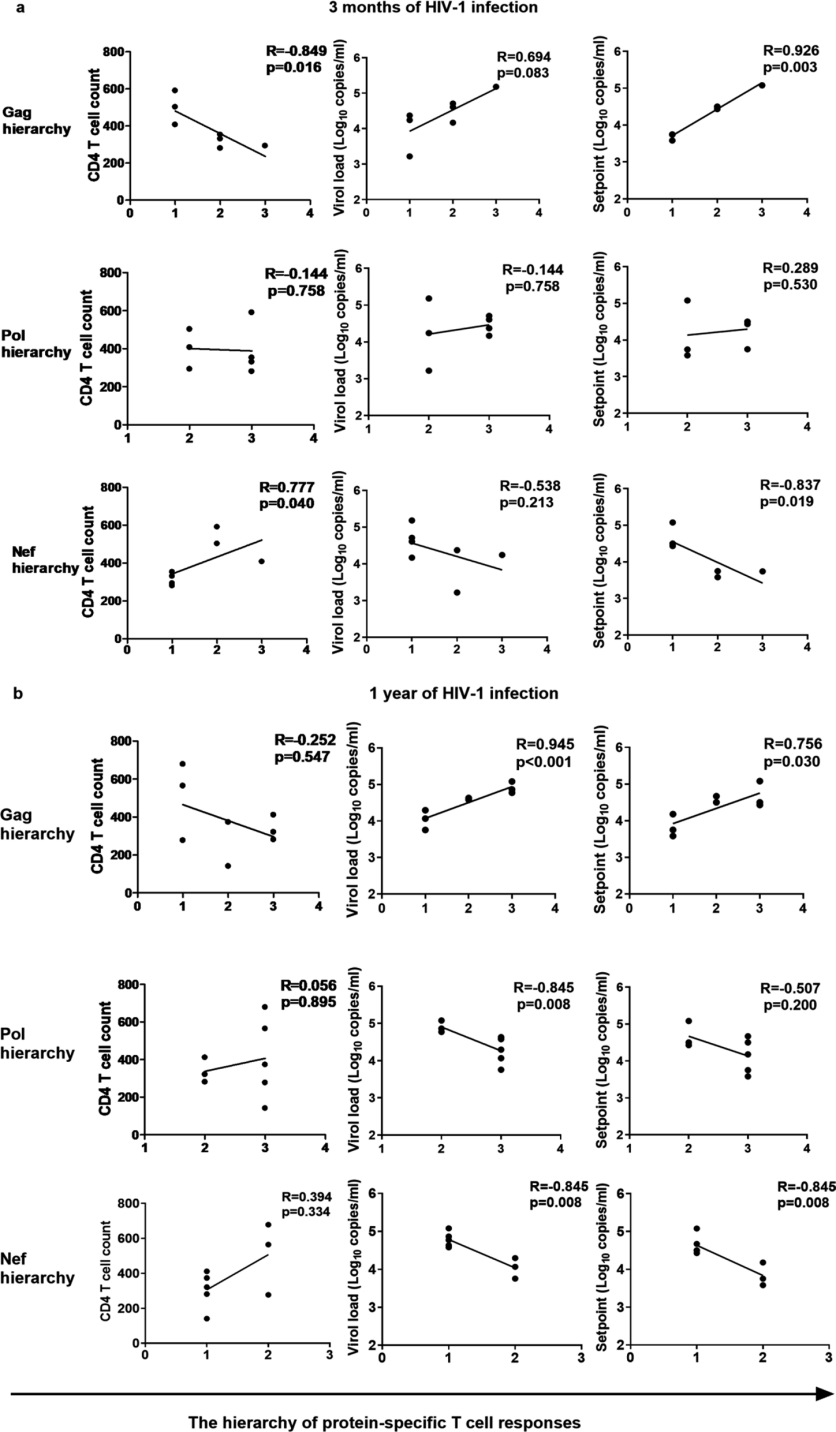
The associations of the hierarchy of Gag-, Pol-, and Nef-specific T-cell responses with disease progression. The hierarchy of Gag-, Pol-, and Nef-specific T-cell responses was assigned the values 1, 2, and 3 based on the strength of protein-specific T-cell response (strong to weak). ﻿Spearman correlations between the hierarchy and CD4 T cell counts, viral loads and viral set points at 3 months of HIV-1 infection (**a**) and at 1 year of HIV-1 infection (**b**) were analyzed

We explored the associations of Gag-, Pol- and Nef-specific T-cell responses with disease progression. The Gag-, Pol-, and Nef-specific responses were assigned the values 1, 2, and 3 based on the strength of protein-specific T-cell response (strong to weak). Subsequently, we analyzed the associations of the response hierarchy with CD4 T-cell counts, VLs, and viral set points, which are reported to be predictors of disease progression [5, 28, 29]. At 3 months of HIV-1 infection, we found that the hierarchy of Gag and Nef protein-specific T-cell responses was correlated with CD4 T-cell counts (*R* =  − 0.849, *P* = 0.016 and *R* = 0.777, *P* = 0.040, respectively), whereas none of the Gag, Pol, and Nef protein-specific T-cell response hierarchy was associated with VLs (*P* > 0.05) (Fig. 2a). Similarly, strong Gag-specific T-cell responses were associated with lower VLs (*R* = 0.945, *P* < 0.001), whereas strong Pol- and Nef-specific responses were associated with higher VLs (*R* =  − 0.845, *P* = 0.008 and *R* =  − 0.845, *P* = 0.008, respectively) at 1 year of infection (Fig. 2b). Moreover, the stronger the Gag- and the weaker the Nef-specific T-cell responses, the lower the viral set point at 3 months of HIV-1 infection (*R* = 0.926, *P* = 0.003 and *R* =  − 0.837, *P* = 0.019, respectively) and at 1 year of HIV-1 infection (*R* = 0.756, *P* = 0.030 and *R* =  − 0.845, *P* = 0.008, respectively) (Fig. 2). This suggests that a higher relative magnitude of Gag-specific T-cell responses might be beneficial for viral control, whereas Nef-specific T-cell responses were associated with rapid disease progression.

### The role of epitope-specific T-cell responses on disease progression

To understand the role of B*13-restricted T-cell responses on disease progression in CRF01_AE subtype HIV-1-infected MSM, we first identified the dynamics of the variations within the B*13-restricted epitopes from the same patients using deep sequencing. Among the 14 patients, 7 acquired sequences at seroconversion, 10 acquired sequences at 3 months of HIV-1 infection, and 12 acquired sequences at 1 year of HIV-1 infection (Tables 1, 2). In total, five fragments containing six epitopes were acquired (Table 2). The mean fragment length was 422 bp. The proportion of bases with a q-score > 30 was 79.9%. On average, 161,775 sequence reads were produced per subject. The variations within RV9 (Nef) epitope were not detected using deep sequencing but by the cloning of sequences due to lack of sample availability. Then, the cross-reactivity of epitope-specific T-cell responses against autologous variant sequences was analyzed in specific individuals. Finally, the associations of the epitope-specific T-cell responses with disease progression were also examined. The detailed results are described below.

**Table 2:** Analysis of longitudinal deep sequencing data for six epitopes in 12 patients

Patients	GI9(Pol)	%	GI11(Gag)	%	VV9(Gag)	%	RI10(Pol)	%	RL9 (Gag)	%	HL9(Gag)	%
	GQDQWTYQI		GQMREPRGSDI		VQNAQGQMV		RQYDQILIEI		RQANFLGRL		HQSLSPRTL	
*320018*												
34dpi	………	100.00	……….	100.00	…….WT	100.00	……V…	100.00	………	100.00	………	100.00
58dpi	………	100.00	……….	100.00	…….WT	100.00	……V…	100.00	………	100.00	………	100.00
330dpi	………	100.00	……….	100.00	…….WT	100.00	……V…	100.00	………	100.00	………	100.00
*320019*												
46dpi	………	100.00	……….	96.90	………	100.00	……S…	100.00	………	100.00	………	100.00
			..I…….	3.10								
58dpi	………	100.00	……….	100.00	………	80.36	……S…	100.00	………	100.00	………	100.00
					……..A	19.64						
381dpi	………	100.00	……….	100.00	………	100.00	……S…	100.00	………	100.00	………	100.00
*320088*												
62dpi	………	100.00	……….	100.00	………	100.00	……P…	86.45	………	100.00	………	100.00
							……S…	13.55				
327dpi	………	100.00	……….	100.00	………	100.00	………	23.28	………	100.00	………	100.00
							……P…	26.90				
							……S…	64.94				
*320135*												
46dpi	………	100.00	..I…….	75.68	………	100.00	………	100.00	………	100.00	..P……	100.00
			……….	24.32								
63dpi	………	100.00	……….	61.47	………	100.00	………	100.00	………	100.00	..PV….	100.00
			..I…….	38.53								
427dpi	………	100.00	……….	100.00	………	100.00	………	100.00	………	100.00	..PV….	100.00
*325020*												
59dpi	………	100.00	……….	70.29	………	64.94	………	98.74	..V……	100.00	…V….	100.00
			..I…….	29.71	…….WT	35.06	……S…	0.91				
							……P…	0.35				
309dpi	………	100.00	……….	91.29	………	57.70	………	100.00	..V……	100.00	…V….	50
			..I…….	8.71	…….WT	42.30					..PV….	31.25
											..LV….	18.75
*440230*												
78dpi	………	100.00	……….	100.00	……..T	100.00	………	100.00	………	100.00	………	100.00
371dpi	………	100.00	……….	100.00	…….WT	65.22	………	100.00	………	100.00	………	100.00
					……..T	34.78						
*300471*												
139dpi	………	100.00	……….	100.00	………	100.00	………	100.00	………	100.00	..PV….	100
360dpi	………	100.00	……….	100.00	………	100.00	……P…	100.00	………	100.00	..PV….	100.00
*320853*												
43dpi	………	100.00	……….	100.00	………	100.00	………	100.00	………	100.00	………	100.00
226dpi	………	100.00	……….	100.00	………	83.95	………	100.00	………	100.00	………	100.00
					…T….	16.05						
*320829*												
51dpi	………	100.00	……….	100.00	………	100.00	………	91.96%	…….K	100.00	..P……	100.00
							……..K	8.04				
120dpi	………	100.00	……….	100.00	………	100.00	………	100.00	…….K	100.00	..P……	100.00
352dpi	………	100.00	……….	100.00	………	100.00	………	100.00	…….KI	66.67	..PV….	100.00
									………	33.33		
*321145*												
38dpi	..G……	61.85	……….	100.00	……..T	100.00	………	100.00	………	100.00	………	100.00
	………	38.15										
138dpi	..G……	100.00	……….	100.00	……..T	100.00	………	100.00	………	100.00	………	100.00
318dpi	..G……	100.00	……….	100.00	……..T	100.00	………	100.00	………	100.00	………	100.00
*321221*												
37dpi	………	100.00	……….	100.00	………	100.00	………	100.00	…….K	100.00	..P……	100.00
393dpi	………	100.00	……….	100.00	………	100.00	………	100.00	…….K	100.00	..PV….	100.00
*325029*												
83dpi	………	100.00	……….	100.00	………	86.61	………	100.00	………	97.90	………	97.77
					…….WT	6.70			…….KI	2.10	..PV….	1.26
					…T….	6.70					..AV….	0.97
337d	………	100.00	……….	100.00	………	100.00	K………	64.60	………	100.00	………	100.00
							………	35.40				

### GI11 (Gag) was conserved and strong T-cell responses were positively associated with CD4 T-cell counts

**Fig. 3:**
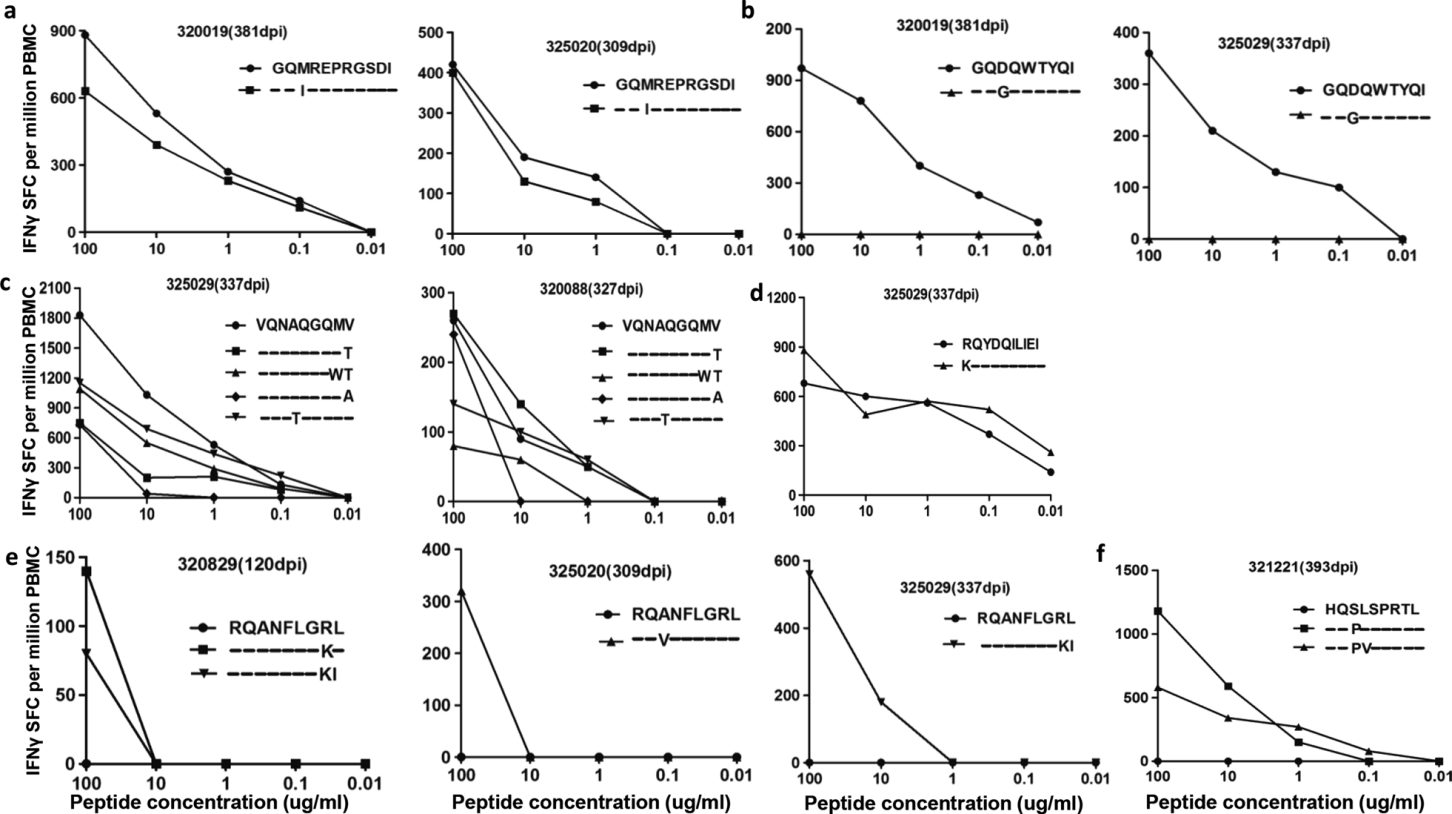
The responses to a panel of variants within epitopes in patients. Cross-reactivity of T-cell responses to GI11 (Gag) (**a**), GI9 (Pol) (**b**), VV9 (Gag) (**c**), RI10 (Pol) (**d**), RL9 (Gag) (**e**), and HL9 (Gag) (**f**) variants in specific individuals

**Fig. 4:**
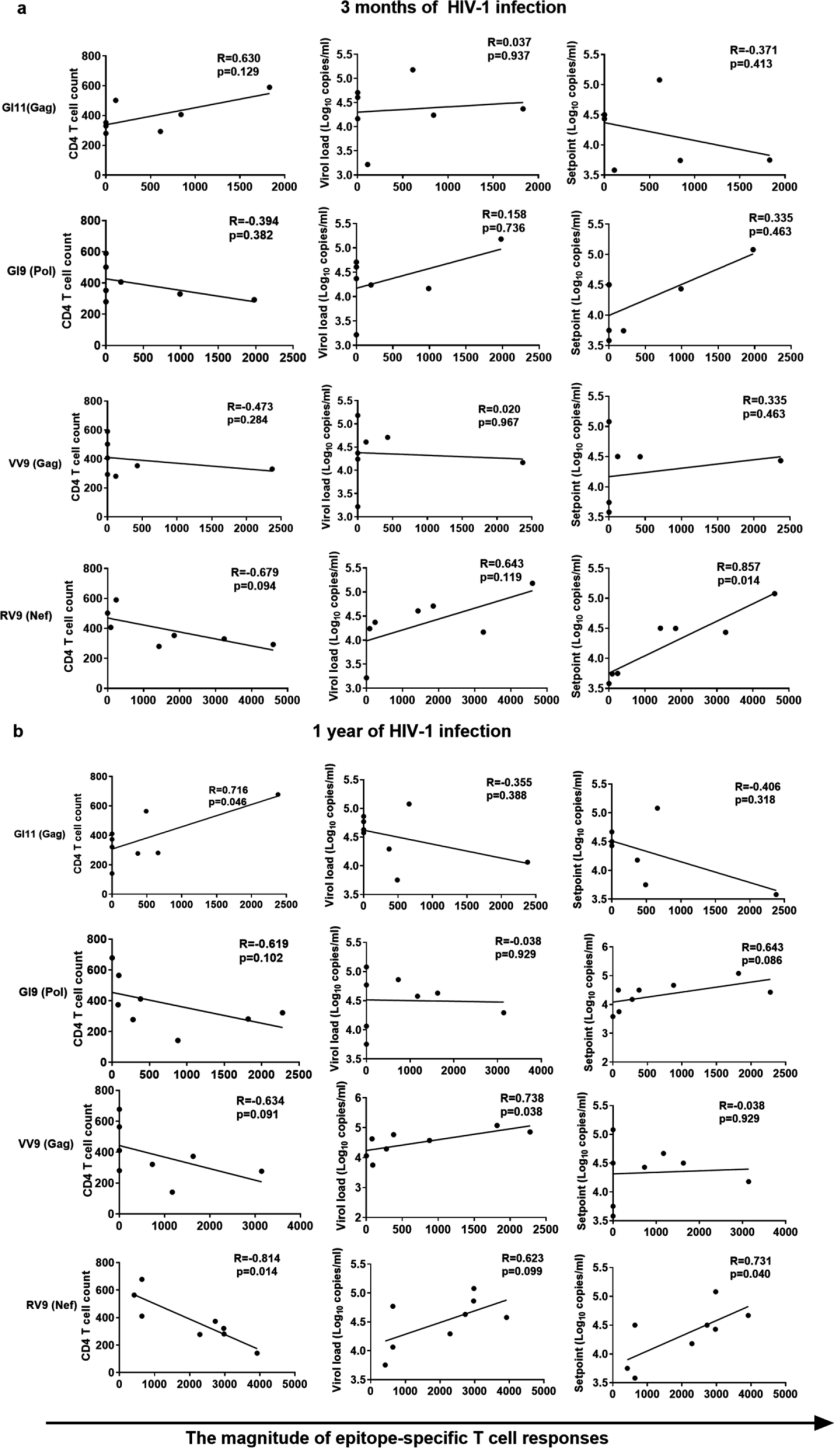
The associations of immunodominant epitope-specific T-cell responses with disease progression. ﻿Spearman correlations between GI11 (Gag), GI9 (Pol), VV9 (Gag) and RV9(Nef) specific T-cell responses with CD4 T cell counts, viral loads and viral set points at 3 months of HIV-1 infection (**a**) and at 1 year of HIV-1 infection (**b**) were analyzed

The GI11 (Gag) epitope was conserved in our study population. Among the 12 patients, three harbored the M228I variant. The M228I variant rates in patients 320135, 325020, and 320019 were 75.68%, 29.71%, and 3.10%, respectively, at seroconversion, decreasing to 38.53%, 8.71%, and 0% at the next follow-up time point. IFN-γ ELISPOT assays were performed for patients 320019 (381 days post-infection (dpi)) and 325020 (309 dpi) whose viral isolates represented the wild-type GI11 (Gag) epitope and the M228I variant (Table 2). The results showed that patients 320019 and 325020 had comparable responses to both epitopes (Fig. 3a). Moreover, no association was observed between GI11 (Gag)-specific T-cell responses with CD4 T-cell counts, VL, or viral set point (Fig. 4a) at 3 months of infection, whereas GI11 (Gag)-specific T-cell responses were positively associated with CD4 T-cell counts (*R* = 0.716, *P* = 0.046) at 1 year of HIV-1 infection (Fig. 4a). These results suggested that the GI11 (Gag) epitope might be protective in B*13-positive CRF01_AE subtype HIV-1-infected MSM, and might be preferred for a T-cell-based vaccine design for the target population.

### GI9 (Pol) was conserved, but GI9 (Pol)-specific T-cell responses were not associated with slow disease progression

The GI9 (Pol) epitope was conserved in our cohort, and only one patient (321145) harbored the D490G variant during follow-up, with a variant rate of 61.85% at 38 days of HIV-1 infection, increasing to 100% at 3 months of HIV-1 infection (Table 2). We synthesized the epitope containing the D490G variant and performed an IFN-γ ELISPOT assay on PBMCs from patients 320019 (381 dpi) and 325029 (337 dpi). The results showed that these two patients had a diminished IFN-γ response to the D490G variant of GI9 (Pol), indicating that the D490G variant represented an escape mutation (Fig. 3b). Furthermore, GI9 (Pol)-specific T-cell responses showed no association with disease progression at either 3 months or 1 year of HIV-1 infection (Fig. 4). Indeed, the emergence of the D490G variant did not affect the CD4 T-cell count or VL in patient 321145. Combined, these results implied that GI9 (Pol)-specific T-cell responses were not associated with slow disease progression.

### The loss of protective VV9 (Gag)-, RI10 (Pol)-, RL9 (Gag)-, and HL9 (Gag)-specific T-cell responses was likely due to the development of escape mutations

The VV9 (Gag) epitope was highly variant. VV9 (Gag)-specific T-cell responses may have promoted the selection of the V143T, V143A, V143T, and M142W/V143T variants in 7 of the 12 patients (Table 2). Specifically, the founder viruses from three patients (320018, 440230 and 321145) carried the M142W/V143T and V143T variants; the V143T variant in patient 320853 and the M142W/V143T variants in patient 325020 were newly developed during follow-up; and the V143A variant in patient 320019 and variants M142W/V143T and V143T in patient 325020 appeared transiently and had disappeared at the next follow-up time point. Moreover, T-cell recognition of VV9 (Gag) variants was detected in patients 325029 (337 dpi) and 320088 (327 dpi), whose predominant viral isolates harbored the wild-type epitope. Patient 325029 showed a strong response to wild-type VV9 (Gag), slightly diminished responses to the A138T and M142W/V143T variants, and weak responses to the V143T and V143A variants. Patient 320088 had similar responses to wild-type VV9 (Gag) and the V143T variant, but showed diminished responses to the A138T, V143A, and M142W/V143T variants (Fig. 3c). The VV9 (Gag)-specific T-cell responses in these two patients showed different cross-reactivity to the variants. In addition, VV9 (Gag)-specific T-cell responses were positively with viral loads at 1 year of HIV-1 infection (*R* = 0.738, *P* = 0.038) (Fig. 4).

The RI10 (Pol) epitope was also highly variant. The I113K, I118V, I118S, I118P, and E121K variations were observed in seven patients (Table 2). The impact of the E121K variant on RI10 (Pol) was assessed in patient 325029 (337 dpi), in whom the predominant viral isolates harbored E121K. The results showed that this variation slightly reduced the RI10 (Pol)-specific T-cell responses (Fig. 3d). We did not evaluate the effects of the other variants on T-cell responses owing to the very low frequency of RI10 (Pol) epitope recognition and the lack of sample availability.

Several variants of the RL9 (Gag) and HL9 (Gag) epitopes were detected in our cohort, such as R436K, L437I, and A431V within RL9 (Gag) and S146P, S146L, S146A, and L147V within HL9 (Gag) (Table 2). Notably, the founder virus from 75% of the study participants carried S146/L147 in HL9 (Gag), which were identified as escape mutations in patients 320829 (120 dpi), 325020 (309 dpi), and 325029 (337 dpi) (Fig. 3e). Similarly, the founder virus from 58.5% of study participants carried the R436/L437 escape mutations in RL9 (Gag), which was identified in patient 321221 (393 dpi) (Fig. 3f) In addition, our study patients responded weakly to HL9 (Gag) and RL9 (Gag). These results suggested that epitopes from founder viruses carrying escape mutations might result in the loss of RL9 (Gag)- and HL9 (Gag)-specific T-cell responses in B*13-positive CRF01_AE subtype HIV-1-infected MSM.

### Early RV9 (Nef)-specific T-cell responses were associated with rapid disease progression

RV9 (Nef)-specific T-cell responses were positively correlated with high viral set points (*R* = 0.857, *P* = 0.014) at 3 months of HIV-1 infection, negatively associated with CD4 T-cell counts (*R* =  − 0.814, *P* = 0.014) and positively correlated with high viral set points (*R* = 0.731, *P* = 0.040) at 1 year of HIV-1 infection (Fig. 4). Moreover, we noticed that the hierarchy of RV9 (Nef)-specific T-cell responses was negatively associated with GI11 (Gag)-specific T-cell responses at 3 months (*R* =  − 0.904, *P* = 0.005) and 1 year (*R* =  − 0.775, *P* = 0.024) of HIV-1 infection, indicating that the patients who displayed high levels of RV9 (Nef)-specific T-cell responses also exhibited low levels of GI11 (Gag)-specific T-cell responses. RV9 (Nef) was also highly variant. The dynamics of the variations within the RV9 (Nef) epitope was studied through the cloning of sequences obtained from seven patients (Table 3). Some variants (R106G/K, Q107K/R/G, E108D, D111G, L112S, and W113R) in the RV9 (Nef) epitope developed in 6/7 patients during follow-up. Consequently, we speculated that escape mutations that appeared early in RV9 (Nef) impaired T-cell responses and were associated with rapid disease progression.

**Table 3:** Longitudinal analyses of variations within the RV9 (Nef) epitope in seven patients

Patient	RQEILDLWV	Clone	Response
*320019*			
58 dpi	………	16/17	1430
	G…….	1/17	
381dpi	………	1/9	640
	.K……	8/9	
*320088*			
62 dpi	………	18/18	3240
327 dpi	………	9/12	2970
	..G……	1/12	
	.R……	1/12	
	.K……	1/12	
*320135*			
63 dpi	..D……	21/21	240
427 dpi	..D……	13/13	420
*325020*			
59 dpi	………	12/14	0
	…..G…	1/14	
	..G……	1/14	
309 dpi	………	14/14	640
*320006*			
108 dpi	………	22/22	90
395 dpi	………	8/12	ND
	……S.	2/12	
	K…….	1/12	
	..K….R	1/12	
*320018*			
58 dpi	………	24/24	4600
330 dpi	………	11/11	2980
*440230*			
78 dpi	.RD……	17/18	ND
	.RD…S.	1/18	
318 dpi	.RD……	8/14	ND
	.GD……	4/14	
	..D……	2/14	

Dpi, days post-infection; ND, not detected

## Discussion

This is the first study to report on the kinetics of B*13-restricted T-cell responses, viral epitope evolution, and their effects on disease progression in CRF01_AE subtype HIV-1-infected MSM during the first year of infection. We found that GI11 (Gag) and GI9 (Pol) were conserved among the three subtypes, but elicited different responses to viral control. The VV9 (Gag), RI10 (Pol), RL9 (Gag), and HL9 (Gag) epitopes were highly variant and displayed no association with disease progression due to the development of escape mutations. Moreover, early RV9 (Nef)-specific T-cell responses were associated with rapid disease progression. This study may increase our understanding of the effects of epitope-specific T-cell responses on disease progression and may help with the design of T-cell-based vaccines for the target population.

The wide distribution of HLA alleles and the presence of differing epidemic HIV-1 strains among different populations lead to differing T-cell response characteristics [14, 15]. Honeyborne and colleagues identified six different B*13-restricted T-cell epitopes in C subtype HIV-1-infected people in Durban, South Africa [19], while GI11 (Gag), VV9 (Gag), and RI9 (Gag) were identified as immunodominant epitopes, while Gag-specific T-cell responses were associated with the control of viremia [19]. B′ subtype HIV-1-infected HLA-A*30–B*13–C*06-positive Chinese individuals have been shown to respond strongly to HL9 (Gag) and RI9 (Gag) epitopes, and these two epitope-specific T-cell responses contribute to slow disease progression [14]. In contrast, in our study, CRF01_AE subtype HIV-1-infected, B*13-positive MSM responded weakly to HL9 (Gag) and RL9 (Gag) (RI9 (Gag) in C and B′ subtype viruses), but responded strongly to RV9 (Nef), GI11 (Gag), GI9 (Pol), and VV9 (Gag). This discrepancy in responses to B*13-restricted epitopes might be due to sequence heterogeneity in the different viral subtypes. For instance, the founder virus for CRF01_AE-infected patients carried A138, R436/L437, and D490 in VV9 (Gag), RL9 (Gag), and GI9 (Pol), respectively, compared with C and B′ subtypes. Additionally, the CRF01_AE founder virus carried S146/L147 when compared with the B′ subtype in the HL9 epitope (HQPISPRTL). The sequences in GI11 (Gag) and RI10 (Pol) were conserved among the three subtypes. This indicated that the variations within the epitopes among the different HIV-1 subtypes contributed to different patterns of immunodominant responses.

Deep sequencing can increase sampling capacity, permit the identification of low-frequency variants, and provide detailed information about early viral dynamics [30–32]. Indeed, low-frequency variants, such as I118S (0.91%)/I118P (0.35%) within RI10 (Pol) in patient 325020 (59 dpi), S146P/L147V (1.26%) and S146A/L147V (0.97%) within HL9 (Gag) in patient 325029 (83 dpi), and M228I (3.10%) within GI11 (Gag) in patient 320019 (46 dpi), were identified in our study. Notably, several low-frequency variant quasispecies disappeared during follow-up, as also previously reported [33, 34]; however, the reason for this disappearance remains unknown. We speculate that the transient emergence of low-frequency variants might affect HIV-1 fitness in vivo. Some mutations within epitopes have been reported to reduce viral replication capacity [35], such as R264K within the B*27-restricted KK10 epitope [36, 37] and T242N within the B*57-restricted TW10 epitope [38, 39]. Only a variant with no fitness cost or multiple mutations that achieve a mutation–fitness balance can become fixed in the viral population [40]. Therefore, exploring the dynamic interplay between T-cell responses and the mechanisms underlying the immune escape of the virus can help inform the type of immunity elicited by T-cell-based vaccines.

T-cell responses to conserved epitopes or highly cross-reactive T-cell responses to variant epitopes have been reported to contribute to slow disease progression in HIV-1-infected patients [14, 41–43]. In our cohort, GI11 (Gag) was conserved, and cross-reactive T-cell responses against the M228I variant were observed. Unlike GI11 (Gag), GI9 (Pol)- and VV9 (Gag)-specific T-cell responses showed different cross-reactivity against variants. In contrast to our results, GI11 (Gag)-specific T-cell responses were reported to be significantly associated with a shorter time to therapy initiation [44]. The inconsistent results of GI11 (Gag)-specific T-cell responses for viral control need validation in a larger population, as well as in vitro.

Nef was the most frequently recognized HIV-1 protein during acute HIV-1 infection with more than 90% of the total HIV-1-specific T-cell responses [45, 46]. It was highly immunogenic and might represent attractive targets for HIV-1 vaccine design. Indeed, the Nef lipopeptides or Nef protein were vaccinated in several clinical trials and Nef-specific T cell responses were detected after vaccination [47–49]. However, the role of Nef-specific T cell responses on disease progression was controversial. Some studies reported that Nef-specific CD8^+^ T cell responses contributed to HIV-1 immune control [50, 51], while others showed that responses to Nef were unlikely to pay a role on disease progression [45, 46, 52–54]. In addition, only 17% of the total virus-specific responses targeting Nef were detected in individuals with chronic HIV-1 infection due to the rapid selection of mutations in T cell epitopes [45]. Nef-specific T-cell responses are driven by a high level of antigenemia, rather than being causal in lowering the VL [55]. In our study, the patients who displayed a high level of RV9 (Nef)-specific T-cell responses also exhibited a low level of GI11 (Gag)-specific T-cell responses. Meanwhile, the rapid development of escape mutations within RV9 (Nef) may contribute to rapid disease progression. This suggests that the competition between the RV9 (Nef) and GI11 (Gag) epitopes for the T-cell repertoire might be one of the mechanisms through which the virus escapes the immune response. Taken together, our results indicate that GI11 (Gag) should be included for T-cell-based vaccine design for HLA-B*13-positive CRF01_AE subtype HIV-1-infected MSM, whereas RV9 (Nef) should be excluded.

Our study had two main limitations. First, we focused on seven well-known, optimal B*13-restricted epitopes located in the Gag, Pol, and Nef proteins of HIV-1 because we did not find any of the new B*13-restricted epitopes in eight patients using the PBMCs at 1 year of infection when we screened from a set of overlapping peptides spanning Gag, Pol, and Nef based on the CRF01_AE subtype consensus sequence [52]. Moreover, the ﻿magnitude and breadth of T cell responses targeting the epitopes were stronger than those targeting the 18-mer peptides, which was in agreement with previous studies [56–58].

However, unidentified B*13-restricted epitopes may exist in other regions of HIV-1, such as Env, Tat and Vpr. Secondly, the dynamics of viral evolution were analyzed using plasma samples collected at a limited number of time points, and we only evaluated the impact of partial variations on T-cell responses. In addition, the number of study patients and samples was small. The corrections for multiple testing were not included for the disease progression analyses. A substantially larger cohort with early HIV-1 infection may help to clarify the role of B*13-restricted epitopes on viral control in greater detail.

## Conclusions

In conclusion, the associations we identified between epitope-specific T-cell responses and disease progression, within-epitope variations, and cross-reactivity of T-cell responses against autologous variant epitopes indicated that GI11 (Gag) was conserved in our cohort. Moreover, strong T-cell responses were positively associated with CD4 T-cell counts, while early RV9 (Nef)-specific T-cell responses were associated with rapid disease progression. GI9 (Pol)-, VV9 (Gag)-, RI10 (Pol)-, RL9 (Gag)-, and HL9 (Gag)-specific T-cell responses did not influence disease progression. Our results strongly suggest that GI11 (Gag) should be included, and RV9 (Nef) excluded, for future T-cell-based vaccine design for HLA-B*13-positive CRF01_AE subtype HIV-1-infected MSM and high-risk individuals.

## Methods

### Ethical approval of the study protocol

The study protocol was approved by the Medical Research Ethics Committee of the First Affiliated Hospital of China Medical University (Shenyang, China). Patients provided written informed consent for blood collection for this study. All methods were carried out in accordance with relevant guidelines and regulations.

### Study population

Fourteen early CRF01_AE subtype HIV-1-infected individuals were recruited from a large-scale, prospective, high-risk MSM cohort in Liaoning, China. This cohort has been previously described [14, 15]. Briefly, HIV-1-negative MSM were followed up every 8 weeks. An enzyme-linked immunosorbent assay targeting HIV specific IgM and IgG (ELISA; Vironostika HIV-1/2 Microelisa System; BioMerieux, Holland) was used to screen for HIV-1 infection, which was further validated by western blotting (HIV Blot 2.2 WB; Genelabs Diagnostics, Singapore). The serum control must appear reactivity against corresponding bands to HIV-1 viral proteins (p17, p24, p31, gp41, p39, p51, p55, p66, gp120 and gp160), the serum control and HIV-2 band. A sample is considered positive if at least two of the three bands coded by the 2 env gene (gp160/gp41 and gp120) and 1gag gene (p17, p24, p55) or 1 pol gene (p31, p51, p66). In the negative control must not appear specific bands or only reactivity to HIV-1 p17. Any viral specific bands present but pattern does not meet criteria for positive is considered indeterminate according to the instructions. The western blotting results at the time of diagnosis were listed at Additional file 1: Table S1. ELISA-negative samples were tested for HIV-1 RNA. Infection was estimated to be 14 days before the date of the RNA-positive and ELISA-negative sampling, or the midpoint of the period between the last negative and first positive results of the ELISA screening tests. All subjects were anti-retroviral naïve during the first year of infection.

### The isolation of peripheral blood mononuclear cells (PBMCs)

Peripheral blood mononuclear cells (PBMCs) were isolated by Ficoll-Paque™Plus (GE Healthcare BioScience, Stockholm, Sweden) density-gradient centrifugation according to manufacturer instructions. Briefly, heparin sodium anticoagulated whole bloods were diluted with 3 × the volume of phosphate-buffered saline (PBS). Carefully layered 35 mL of diluted cell suspension over 15 mL of Ficoll-Paque in a 50 mL conical tube and then centrifuged at 400 × *g* for 30 min in a swinging-bucket rotor without brake. Aspirated the upper layer leaving the mononuclear cell layer undisturbed at the interphase and then carefully transferred the mononuclear cell layer to a new 50 mL conical tube. Washed the cells twice with PBS by centrifuging cells at 300 × *g* for 10 min. Cryopreserved in the frozen stock solution with 10% DMSO and 90% FBS and stored in liquid nitrogen until use.

### CD4 T-cell counts and detection of viral load (VL)

The number of CD4^+^ T-cells was determined using a FACSCalibur Flow Cytometer (Becton Dickinson, Franklin Lakes, NJ, USA). The number of HIV-1 RNA copies, i.e. the VL, was determined using the COBAS AmpliPrep/COBAS TaqMan HIV-1 Test assay (Roche, Basel, Switzerland). The viral set point was defined as the average VL (≥ 3 time points) from 120 days until 1 year of infection.

### HLA typing

DNA was extracted from anticoagulated whole blood using the QIAamp™ Blood DNA Mini Kit (Qiagen, Stanford, VA, USA) according to the manufacturer’s protocol. Two-digit HLA typing was performed with the PCR–sequence-specific primer method (One Lambda, Los Angeles, CA, USA).

### Synthetic peptides

Seven known B*13-restricted epitopes (four in the Gag protein: GQMREPRGSDI (Gag_226–236_, GI11), VQNAQGQMV (Gag_135–143_, VV9), RQANFLGRL (Gag_429–437,_ RL9), and HQSLSPRTL (Gag_144–152_, HL9); two in the Pol protein: GQDQWTYQI (Pol_487–498_, GI9) and RQYDQILIEI (Pol_113–122_, RI10); and one in the Nef protein: RQEILDLWV (Nef_106–114_, RV9)) were synthesized by Sigma–Aldrich (Saint Louis, MO, USA), six of which were from the lists of best-defined HIV CTL/CD8^+^ epitopes (A-list, https://www.hiv.lanl.gov/content/immunology/tables/optimal_ctl_summary.html) [19, 59]. The B*13-restricted HL9 which was newly identified and showed to be associated with slow disease progression by our previous study [14] was also included in the analysis. This set of peptides was designed based on the consensus sequence of ~ 50 near-full-length sequences from the same cohort with acute CRF01_AE infection [52]. Twelve additional epitopes harboring amino acid variants present in the study population that differed from the CRF01_AE-associated consensus sequence were synthesized by GL Biochem (Beijing, China).

### Interferon-gamma (IFN-γ) enzyme-linked immunospot (ELISPOT) assays

The IFN-γ ELISPOT assay was performed as previously described [14]. Briefly, 96-well plates were precoated with an anti-IFN-γ monoclonal antibody at 4 ℃ overnight. A total of 100,000 cells per well were plated (BD Biosciences, San Jose, CA, USA) with epitope (5 μg/mL). Phytohemagglutinin (10 μg/mL) was used as a positive control, and medium alone served as the negative control. The plates were incubated for 20 h at 37 °C in a humidified atmosphere with 5% CO_2_. The spots were counted with the ImmunoSpot® Analyzer (Cellular Technology, Shaker Heights, OH, USA). The results were expressed as spot-forming cells (SFC) per 1 × 10^6^ PBMCs. Responses were considered positive if there were at least three-fold more SFCs than the mean number of SFCs in the negative control, as well as at least 50 SFCs per 1 × 10^6^ PBMCs.

### Deep sequencing of viral RNA

HIV-1 RNA was extracted from plasma samples using the QIAamp Viral RNA Mini Kit (Qiagen, Hilden, Germany) and reverse transcribed to cDNA using the SuperScript III Kit (Life Technologies, Grand Island, NY, USA) with random hexamers. cDNA fragments were amplified by two rounds of nested PCR using the KOD-Plus-Neo Kit (Toyobo, Osaka, Japan). The fragments and primers are listed in Additional file 2: Table S2.

The first round of PCR with outer primers was performed under the following conditions: 94 °C for 5 min; 30 cycles of 94 °C for 30 s, 55 °C for 30 s, and 68 °C for 3 min 30 s; and a final extension step at 68 °C for 10 min. For the second round of PCR with inner primers for fragments containing the GI11 (Gag) epitope, the following conditions were used: 94 °C for 5 min; 30 cycles of 94 °C for 30 s, 45 °C for 30 s, and 68 °C for 3 min 30 s; and a final extension step at 68 °C for 10 min. For fragments containing the GI9 (Pol), VV9 (Gag), RI10 (Pol), RL9 (Gag), or HL9 (Gag) epitopes, the conditions for the second round of PCR were as follows: 94 °C for 5 min; 30 cycles of 94 °C for 30 s, 55 °C for 30 s, and 68 °C for 3 min 30 s; and a final extension step at 68 °C for 10 min.

cDNA library construction and sequencing were as in our previous study [60]. Briefly, the PCR products were purified using Agencourt AMPure XP beads (Beckman Coulter, Brea, CA, USA) and quantified using a Qubit 3.0 Fluorometer (Life Technologies, Carlsbad, CA, USA). The PCR products were then indexed with an adaptor using a TruSeq Nano DNA LT Library Preparation Kit (Illumina, San Diego, CA, USA). The indexed DNA libraries were quantified using a LightCycler® 480 (LC480) Real-Time PCR system (Roche), pooled, denatured, and mixed with 50% PHIX Control Libraries (Illumina). Deep sequencing was undertaken using an Illumina MiSeq System. DADA2 software was used to analyze the representative sequences for each fragment (DADA2: High-resolution Sample Inference from Illumina Amplicon Data). A threshold of > 0.1% was used for reporting variants.

### Cloning and sequencing of the HIV-1 nef gene

The entire nef gene was amplified using the SuperScript Polymerase One-Step RT-PCR System (Takara, Dalian, China) followed by nested PCR. The outer primers were O1 (5′-GTGCCTCTTCAGCTACCACCG-3′, 8513–8533 of HXB2) and O2 (5′-AGCATCTGAGGGTTAGCCACT-3′, 9488–9508 of HXB2). The first round of PCR was performed with the following parameters: 56 °C for 30 min; 94 °C for 5 min; followed by 3 cycles of 94 °C for 30 s, 50 °C for 30 s, and 72 °C for 2 min and 32 cycles of 94 °C for 30 s, 55 °C for 30 s, and 72 °C for 2 min; and a final extension step at 72 °C for 10 min. The inner primers were I1 (5′-TGGACAGATAGGGTTATAGAA-3′, 8697–8717 of HXB2) and I2 (5′-CACCTCCCCTGGAAAGTCCCC-3′, 9448–9468 of HXB2). The conditions for the second round of PCR were as follows: 94 °C for 5 min; 3 cycles of 94 °C for 30 s, 50 °C for 30 s, and 72 °C for 2 min and 32 cycles of 94 °C for 30 s, 55 °C for 30 s, and 72 °C for 2 min; and a final extension step at 72 °C for 10 min. The PCR products were purified using the QIAquick Gel Extraction Kit (Qiagen) and cloned using a TOPO TA cloning kit (Invitrogen, USA). The fragments were sequenced by Huada Genomics Company (Beijing, China). The Sequencher program (version 4.9) was used to assemble and edit the sequence fragments.

### Statistical analyses

Spearman’s correlation was used to investigate the relationship between T-cell responses and the clinical parameters of HIV-1 (CD4 T-cell counts, VLs, and viral set point). *P* < 0.05 was considered significant. Data were analyzed using SPSS v17.0 (IBM, Armonk, NY, USA). Graphs were created using GraphPad Prism v5.0 (GraphPad Software, San Diego, CA, USA).

## Supplementary Information

**Additional file 1. Table S1.** Information on western blotting results of all patients at the time of diagnosis. **Table S2.** Information on the primers used in deep sequencing of HIV RNA

## Data Availability

The datasets generated and/or analyzed as part of this study are available in the NCBI repository, [Submission number: PRJNA807734 for next generation reads and BankIt2552230 (OM799324-OM799542) for clone sequences]. Any additional information may be requested by contacting the corresponding author on reasonable request.

## Abbreviations

HIV: Human immunodeficiency virus
HLA: Human leukocyte antigen
MSM: Men who have sex with men
ELISA: Enzyme-linked immunosorbent assay
ELISPOT: Enzyme-linked immunospot
VL: Viral load
PBMCs: Peripheral blood mononuclear cells
SFC: Spot-forming cells
dpi: Days post-infection
