# Supplementary material for: A longitudinal analysis of immune escapes from HLA-B*13-restricted T-cell responses at early stage of CRF01_AE subtype HIV-1 infection and implications for vaccine design
Source: BMC Immunol. 2022 Apr 2;23:15. doi: 10.1186/s12865-022-00491-7 (PMC8976269; doi:10.1186/s12865-022-00491-7)
Supplement: Supplementary file 1 — Additional file 1. Table S1. Information on western blotting results of all patients at the time of diagnosis. Table S2. Information on the primers used in deep sequencing of HIV RNA [file 12865_2022_491_MOESM1_ESM.docx]

**A longitudinal analysis of immune escapes from HLA-B*13-restricted T-cell responses at early stage of CRF01_AE subtype HIV-1 infection and implications for vaccine design**

Hui Zhang^1,2,3,4^, Chuan He^1,2,3,4,5^, Fanming Jiang^1,2,3,4,5^Shuang Cao^1,2,3,4,6^, Bin Zhao^1,2,3,4,^ Haibo Ding^1,2,3,4^, Tao Dong^7,8^, Xiaoxu Han^1,2,3,4*^, Hong Shang^1,2,3,4*^

^1^NHC Key Laboratory of AIDS Immunology (China Medical University), National Clinical Research Center for Laboratory Medicine, The First Affiliated Hospital of China Medical University, Shenyang 110001, China

^2^Key Laboratory of AIDS Immunology, Chinese Academy of Medical Sciences, Shenyang 110001, China

^3^Key Laboratory of AIDS Immunology of Liaoning Province, Shenyang 110001, China

^4^Collaborative Innovation Center for Diagnosis and Treatment of Infectious Diseases, 79 Qingchun Street, Hangzhou, 310003, China

^5^Department of Laboratory Medicine, The First Affiliated Hospital of China Medical University, Shenyang, 110001, China

^6^Department of Laboratory Medicine, China Medical University Shengjing Hospital Nanhu Branch, Shenyang 110001, China

^7^Chinese Academy of Medical Sciences Oxford Institute, Nuffield Department of Medicine, Oxford University, United Kingdom

^8^Medical Research Council Human Immunology Unit, Weatherall Institute of Molecular Medicine, John Radcliffe Hospital, Oxford University, United Kingdom

**Correspondence**

Hong Shang, NHC Key Laboratory of AIDS Immunology (China Medical University), National Clinical Research Center for Laboratory Medicine, The First Affiliated Hospital of China Medical University, 155 Nanjing North Street, Heping District, Shenyang, Liaoning Province, 110001, China. Telephone: +86 (24) 8328-2634; fax: +86 (24) 8328-2634; email: [hongshang100@hotmail.com](mailto:hongshang100@hotmail.com).

Xiaoxu Han, NHC Key Laboratory of AIDS Immunology (China Medical University), National Clinical Research Center for Laboratory Medicine, The First Affiliated Hospital of China Medical University, 155 Nanjing North Street, Heping District, Shenyang, Liaoning Province, 110001, China. Telephone: +86 (24) 8328-2634; fax: +86 (24) 8328-2634; email: xxhan@yeah.net.

Additional file 1: Table S1. Information on western blotting results of all patients at the time of diagnosis

Table S1. Information on western blotting results of all patients at the time of diagnosis

| PID | dpi^a^ | Band profile |
| --- | --- | --- |
| 320829 | 43 | gp160, p24 |
| 320018 | 31 | gp160, gp120, p24 |
| 320853 | 43 | gp160, gp120, p24 |
| 321145 | 45 | gp160, gp120, p 24, p17 |
| 320019 | 41 | gp160, gp120, p24 |
| 321221 | 29 | gp160, gp120, p24 |
| 320088 | 62 | gp160, gp120, p24 |
| 300471 | 139 | gp160, gp120, p66, p55, p51, gp41, p39, p31, p24, p17 |
| 325020 | 59 | gp160, gp120, p24 |
| 325029 | 27 | gp160, gp120, gp41, p24 |
| 320571 | 65 | gp160, gp120, p66, p55, p51, gp41, p39, p31, p24, p17 |
| 320006 | 26^b^ | gp160, p24 |
|  | 39^b^ | gp160, p24, p17 |
|  | 81^b^ | gp160, p24, p17 |
|  | 108 | gp160, gp120, p66, p55, p51, gp41, p39, p31, p24, p17 |
| 320135 | 52 | gp160, gp120, p66, p55, p51, gp41, p31, p24, p17 |
| 440230 | 71 | gp160, gp120, p66, p51, gp41, p31, p24, p17 |

dpi, days post-infection;

^a^ days post-infection at the time of diagnosis

^b^ Tested indeterminate to HIV at 26, 39 and 81 dpi.

Additional file 1: Table S2. Information on the primers used in deep sequencing of HIV RNA

Table S2. Information on the primers used in deep sequencing of HIV RNA

| Epitope | HXB2 position | Amplicon | Outer primer | Inner primer |
| --- | --- | --- | --- | --- |
| VV9(Gag)  HL9(Gag) | Gag(135−143)  Gag(144−152) | 1074−1500 | F: ATCTCTAGCAGTGGCGCCCGAACAG  R: TAATGCTTYTATTTTYTCTTYTGTYAATGGC | F: WGAYACCAARGAAGCYTTAGA  R: GTTCCTGCTATRTCACTWCCCCTYGGTTC |
| GI11(Gag) | Gag(226−236) | 1321−1752 | F: ATCTCTAGCAGTGGCGCCCGAACAG  R: TAATGCTTYTATTTTYTCTTYTGTYAATGGC | F: GGAGCCACCCCACAAGATTTAAA  R: CAAGGTTTCWGTCATCCARTTTTT |
| RL9(Gag) | Gag(429−437) | 1817−2281 | F: ATCTCTAGCAGTGGCGCCCGAACAG  R: TAATGCTTYTATTTTYTCTTYTGTYAATGGC | F: TAGAAGARATGATGACAGCATG  R: AGGGGTCGTTGCCAAAGAGTGA |
| RI10(Pol) | Pol(113−122) | 2376−2833 | F: TGGAAATGTGGRAARGARGGAC  R: CCTGTHTGCAGHCCCCAATATGTT | F: TGGAAACCAAAAATGATAGGGG  R: GGTATTCCTAATTGAACTTCCCA |
| GI9(Pol) | Pol(488−496) | 3300−3703 | F: TGGAAATGTGGRAARGARGGAC  R: CCTGTHTGCAGHCCCCAATATGTT | F: AGCTGGACTGTCAATGATATAC  R: TTTCCCCATATTACTATGCT |

F, forward; R, reverse.
